# Exploring the publication gap in pediatric randomized clinical trials: completed vs. uncompleted pediatric clinical trials

**DOI:** 10.3389/fmed.2025.1590125

**Published:** 2025-05-30

**Authors:** Katerina Nebeska, Lenka Souckova, Radka Stepanova, Regina Demlova

**Affiliations:** ^1^Faculty of Medicine, Centre of Excellence CREATIC, Masaryk University, Brno, Czechia; ^2^European Clinical Research Infrastructure Network, Paris, France; ^3^Department of Pharmacology, Faculty of Medicine, Masaryk University, Brno, Czechia; ^4^International Centre for Clinical Studies, St. Anne’s University Hospital in Brno, Brno, Czechia

**Keywords:** registry, clinical trials, pediatrics, results, publication bias

## Abstract

**Background:**

Randomized clinical trials (RCTs) are essential for advancing medical knowledge, especially in pediatrics, where they provide critical evidence for safe and effective treatments. Regulatory frameworks like the Pediatric Regulation in the EU and the Best Pharmaceuticals for Children Act in the U.S. have spurred growth in pediatric RCTs. However, these clinical trials face unique challenges, including ethical complexities, recruitment difficulties, and funding limitations, which can hinder their completion. The publication of RCTs’ results is equally crucial, yet not all completed RCTs share their findings, contributing to publication bias. This bias, especially in pediatric clinical trials, can distort medical evidence, impact clinical decisions, and potentially compromise patient safety.

**Methods:**

We conducted a cross-sectional analysis of pediatric RCTs registered on ClinicalTrials.gov between 2011 and 2013, with completion status by 2017. Inclusion criteria included clinical trials with drug interventions in participants aged 0-17 and randomization. Data on RCTs characteristics, including phase, funding source, participant age, and enrollment, were extracted. RCTs completion status was assessed, and reasons for incompletion were categorized. Publication status was evaluated through registry and manual searches on PubMed and Google Scholar. Statistical analyses, including logistic regression, were performed to identify factors associated with trial incompletion and non-publication.

**Results:**

Out of 2875 pediatric clinical trials reviewed, 1088 met the inclusion criteria. Among these, 16.54% were uncompleted, primarily due to patient accrual issues (32.22%). Academic sponsors funded 48.53% of trials, and industrial sponsors funded 45.13%. Of 908 completed RCTs, 58.48% posted results in registries, while 70% had results published in peer-reviewed journals. Industrially funded RCTs were more likely to post results, but academic RCTs had a higher scientific publication rate. The median time to first result publication in registries was 21 months, with significant delays linked to the trial phase, funding, and participant enrolment size.

**Conclusion:**

Our study highlights significant challenges in pediatric RCTs, including high incompletion rates and delays in result reporting. Ethical, regulatory, and logistical barriers hinder progress, impacting evidence transparency. Strengthened regulatory oversight and enhanced compliance are essential to improve pediatric research outcomes and ensure timely dissemination of RCTs results.

## 1 Introduction

Randomized clinical trials (RCTs) are pivotal in medical research and healthcare as their rigorous design minimizes bias and delivers high-quality evidence on the safety and efficacy of new medicines. The same prerequisites apply to RCTs in pediatrics. In most cases, new pediatric medicines are launched to the market only after thorough evaluation through RCTs, which remain the gold standard for the Regulatory Agencies.

Complementing new drug applications, pediatric RCTs also form the cornerstone of evidence-based medicine (EBM) when professional societies, medical practitioners, and policymakers rely on the results of well-conducted RCTs to set up or update guidelines, make informed decisions about patient care, and adjust healthcare policies. Investigator-initiated trials (IITs) aim to answer unresolved questions from clinical practice by conducting RCTs to gather more robust evidence on frequent off-label drug use in pediatrics. This approach is particularly beneficial in pediatrics, where IITs are strongly represented.

To launch the new medicine for pediatric patients to the market, to optimize the standard pediatric health care, and to adjust the clinical guidelines appropriately, the first assumption is (i) the sufficient number of pediatric clinical trials, the second prerequisite is (ii) their completion and the third crucial condition is the (iii) open access to their results.

### 1.1 Trends in pediatric RCTs

There was a significant increase in pediatric research in Europe after introducing the Pediatric Regulation (EC) No 1901/2006 (1). The proportion of clinical trials that include children increased by 50% between 2007 and 2016, from 8.25 to 12.4%, leading to more evidence-based information on medicines used in children (2).

In the U.S., the Best Pharmaceuticals for Children Act (BPCA) of 2002 and the Pediatric Research Equity Act (PREA) of 2003 established incentives and mandates for pediatric drug development, respectively. Since 2007, FDA has issued priority review vouchers for tropical diseases, medical countermeasures, and rare pediatric diseases, and this voucher program has been extended for rare disease product applications with a rare pediatric disease designation through September 30, 2024 (3).

### 1.2 Proportion of the uncompleted pediatric RCTs

Despite regulatory support, initiating and conducting trials in the pediatric population is still more difficult than in adults because of multifaceted ethical barriers, the overall low burden of disease, unfavorable market incentives, and lack of funding (4). Examining the number of pediatric RCTs conducted over a specific period would provide valuable insights.

Ethically, enrolling minors in clinical research demands strict protective measures, parental consent, and assent depending on age, which can complicate participant recruitment and retention (5). Moreover, the smaller size of pediatric populations for certain conditions makes reaching adequate sample sizes difficult, affecting the clinical trial’s statistical power and potential completion. Some of these barriers may lead to delay or incompletion of clinical trials and thus to waste unnecessarily included pediatric patients.

Previous work has demonstrated that 11% of interventional pediatric RCTs registered in ClinicalTrials.gov during the years 2007-2020 were prematurely discontinued, with poor participant recruitment as a predominant reason for trial incompletion (4). This issue could have ethical, social, health and economic implications: patients consent to participate in the trial with the expectation that the clinical findings will foster new medical knowledge. Therefore, trial incompletion may undermine patients’ trust in clinical research and represent a waste of financial (public) and human resources (6).

### 1.3 Release of the RCT’s results

According to the Declaration of Helsinki, it is the responsibility of the researchers and sponsors to disseminate the trial results involving human participants, regardless of the findings. In the EU, according to EC guideline 2012/c302/03, the sponsor must disclose the results of the registered trial in EUCTR to EMA within 12 months of the trial completion. In the case of a pediatric clinical trial, the timeline is shortened to 6 months (7). In addition, Regulation (EU) No 536/2014 mandates that a summary of the results for the lay public accompanies the clinical trial results.

For trials with at least one U.S. site, a 2007 FDA amendment requires that results be posted on ClinicalTrials.gov within 1 year of clinical trial completion. Moreover, in September 2016, the U.S. Department of Health and Human Services issued a Final Rule for Clinical Trials Registration and Results Information Submission (42 CFR Part 11) that clarifies and expands the regulatory requirements and procedures for submitting registration and summary results information of clinical trials on ClinicalTrials.gov, under FDAAA 801. Trial results published in registries should include a summary of participants’ information, protocol and changes, summary results for pre-specified primary and secondary endpoints, details of adverse events, and statistical analyses (8).

### 1.4 Risk and implications of publication bias in pediatric RCTs

Although the abovementioned regulations have been in force for a long time, their compliance by clinical trial sponsors is not fully respected (9, 10). The non-publication of results from pediatric clinical trials is a critical issue with multiple underlying causes and significant consequences. One of these consequences is the phenomenon of publication bias, where trials with negative or inconclusive results are less frequently published, and this phenomenon is well documented in medical research (11). In pediatrics, the risk of publication bias is elevated due to the unique dependency on clinical trials for determining appropriate treatments for children. Many medications used in pediatric care do not have specific approval for use in children and are instead prescribed off-label (12). This practice makes the accurate and comprehensive reporting of clinical trial results crucial, as it directly influences the safety and efficacy of medicines administered to this vulnerable population. The lack of publication of completed and unbiased clinical trial results may lead to an incomplete understanding of a medication’s risk profile, potentially resulting in adverse effects that could have been mitigated or avoided.

The impact of non-publication of clinical trial results extends beyond academic research and has implications for clinical decision-making and policy-making (6). By understanding and addressing the causes of clinical trial incompletion and non-publication, researchers and policymakers can enhance the reliability and accessibility of medical evidence, ensuring that the benefits of pediatric clinical trials are fully realized and contribute to evidence-based medicine.

## 2 Materials and methods

The aim of this research work was firstly to map the current trends of a number of pediatric clinical trials, their rate of completion, and their results availability. As a second aim, this cross-sectional, data-based analysis focuses on exploring the factors leading to the high rates of incompletion and non-publication of pediatric clinical trial results and offering insights that could potentially improve future research practices and policy interventions.

### 2.1 Data source

We systematically searched randomized pediatric clinical trials, which were registered on ClinicalTrials.gov. ClinicalTrials.gov is an online database of clinical research studies that contains more than 490,000 studies from 222 countries, making it the most comprehensive WHO-recognized registry (13). The clinical trials’ selection was done in this database according to predefined inclusion criteria:

1. RCT,
2. pediatric (the age of participants was limited from birth to 17 years),
3. with the drug intervention,
4. registered between 1.1.2011 and 31.12.2013,
5. completed or uncompleted by 31.12.2017.

The recruitment status of clinical trial participants has, therefore, been narrowed down to completed, withdrawn, suspended, and terminated according to ClinicalTrials.gov Glossary Terms (14). According to this glossary, a trial was considered completed if it had ended normally and participants were no longer being examined or treated. If the trial stopped early, before the first participant was enrolled, we refer to it as withdrawn. The trial was classified as suspended if it had stopped early, but could start again. The trial was given a terminated status if it was stopped prematurely and not restarted. Participants were no longer examined or treated. The content we searched was downloaded in JSON format on the same day as our search for detailed analysis, ensuring the file’s immutability.

The exclusion criteria follow:

1. non-randomized,
2. non-pediatric,
3. without drug intervention,
4. without final confirmed status after December 31, 2017,
5. trial registration was done > 60 days after the clinical trial start date (15).

The trial was included in our analysis if it fulfilled all the inclusion criteria and did not meet any of the exclusion criteria. This research is an independent follow-up survey to the work of Harvard Medical School researchers Pica and Bourgeois (16), whose work was completed with pediatric RCTs registered by the end of 2010. The timeframe chosen for the pediatric clinical trials included in the registry is therefore linked to Pica’s research, while allowing sufficient time for the trials to be completed and the results to be published.

The EU Clinical Trials Register (EUCTR) was consulted to identify the trial identifier (EudraCT number) and to determine whether trial results had been published in the registry. This procedure has only been used for studies taking place in the EU. PubMed and Google Scholar were also systematically searched to identify relevant publications in peer-reviewed journals.

### 2.2 Clinical trial characteristics

We collected the following trials’ specific data from the ClinicalTrial.gov downloaded content to provide pediatric RCTs characteristics:

a) Year of RCT registration;

– 2011, 2012, 2013

b) Age of participant;

– Clinical trial participants were categorized by age into “preterm, newborn, infant,” “toddler and preschool,” “school age,” “adolescent,” “mixed ages,” and “combined.” While the “mixed ages” clinical trials include children of different ages, the “combined” clinical trials include children and adults.

c) Funding source (academic, industry, other);

– ClinicalTrial.gov describes the funder as an organization that provides funding and support for a clinical trial (including activities such as design, data analysis, and reporting). Both sponsors and collaborators are considered as funders according to the registry. ClinicalTrials.gov distinguishes funding types as industry, the National Institutes of Health (NIH), and government agencies (other than NIH) and other. We have categorized the funding source based on sponsor and collaborator, similar to what has been described in other trials (4, 16, 17). When only the sponsor was mentioned in the ClinicalTrials.gov registry, we considered it the funder. If more than one sponsors were listed, we determined the lead sponsor to be the primary funder (16).

– Subsequently, we used three funder categories: academic, industry, and other. While trials where hospitals, universities, and foundations were listed as sponsors, we classified them as academic funders, trials with sponsors from the industry (pharmaceutical companies, any profit organization) were assigned to industry funders. Category other included all government-funded trials.

d) Masking;

– Open-label, single, double, triple, quadruple

e) Actual enrollment;

– ClinicalTrials.gov allows for the identification of the number of patients planned for the trial and the number of patients recruited.

f) Sample size;

– For each of these patients’ groups, we created 5 sample sizes: 0-50, 51-100, 101-500, 501-1000, and > 1000.

g) trial phase;

– I, II, III, IV

h) medical condition;

– Medical conditions were classified according to the Internal Classification of Diseases (ICD-10) (18).

i) the justification for an incompletion;

– In the case of uncompleted clinical trials, we ascertained the reason for termination, which was stated directly in the ClinicalTrials.gov registry or the published article, if any. Reasons for termination were categorized as follows: patient accrual, company/business decision, informative termination, funding or regulatory issue, conduct problems, principle investigator left and non-reported or unclear reason.

### 2.3 Publication related to pediatric RCTs’ results search

The search for publications of pediatric RCT results in peer-reviewed scientific journals followed a structured approach:

1st step: Registry Search

j) We first searched for publications directly in the registry for each included pediatric RCT. Any publications found were reviewed to assess their relevance to the clinical trial. A publication was considered relevant if it mentioned the ClinicalTrials.gov Identifier (NCT) of the completed or terminated pediatric RCT in either the article’s abstract or full text, and if it was published after the clinical trial’s completion.

2nd step: Manual Search

k) For pediatric RCTs with no publications or non-relevant publications listed in the registry, two independent researchers (KN and LS) conducted additional searches on PubMed and Google Scholar. The search was based on the trial’s NCT or EudraCT number, trial title, author names, institutions, and clinical trial keywords.

3rd step: Publication Linkage

l) Articles were linked to the respective trial by comparing trial data from the registry with information in the abstract or full manuscript when needed. In cases where multiple publications were identified for a single trial, we selected the earliest publication.

#### 2.3.1 Time to publication

We decided to search pediatric RCTs completed by December 31, 2017, allowing investigators at least 2 years to analyze data, draft a manuscript, and complete the necessary steps for publication in peer-reviewed journals (19). This extended timeframe was intended to provide sponsors and researchers sufficient time to publish their trial results.

m) The time to publication was calculated by determining the number of months between the trials’s completion and the publication date. The trial completion date was obtained from ClinicalTrials.gov, while the publication date was taken from the first available version, either in print or electronic format.

### 2.4 Release of pediatric RCTs results search

n) The ClinicalTrials.gov registry was used to search for the results of completed trials that met the inclusion criteria. Trials without published results in ClinicalTrials.gov were searched in the EU Clinical Trial Register (EUCTR). If the trials’ results were published in the EUCTR, we added them to the trials with found results.

### 2.5 Data quality control checking

An independent researcher (RS) controlled each 10th trial. Each data point for all included RCTs was verified against the source information in the registry. In case of discrepancies or missing data, the researchers were informed, and any discrepancies were resolved through discussion and mutual agreement. Corrections were made where necessary following consensus.

### 2.6 Statistical analysis

All statistical analyses were performed using SAS version 9.4 (SAS Institute Inc., Cary, NC, United States). Descriptive statistics were used to summarize both categorical and continuous variables. For categorical variables, data were presented as counts (n) and percentages (%), with graphical representations where appropriate. For continuous variables, results were reported using the median, along with the minimum and maximum values. A chi-square test was applied to compare categorical variables, and *P*-values were reported to indicate statistical significance. A *P*-value of < 0.05 was considered statistically significant.

To find factors influencing a publication gap, a two-step approach was employed to build the final multivariate logistic regression model. Univariate logistic regression was performed for each independent variable to assess its association with the dependent variable. All variables with a *P* < 0.1 in the univariate analysis were selected for inclusion in the multivariate logistic regression model. Only the significant factors (i.e., parameters with *P* < 0.05) from multivariate models are presented in tables. The results of the logistic regression are presented as odds ratios (OR), along with 95% confidence intervals (CI) and *P*-values.

## 3 Results

### 3.1 Trial characteristics

**FIGURE 1:**
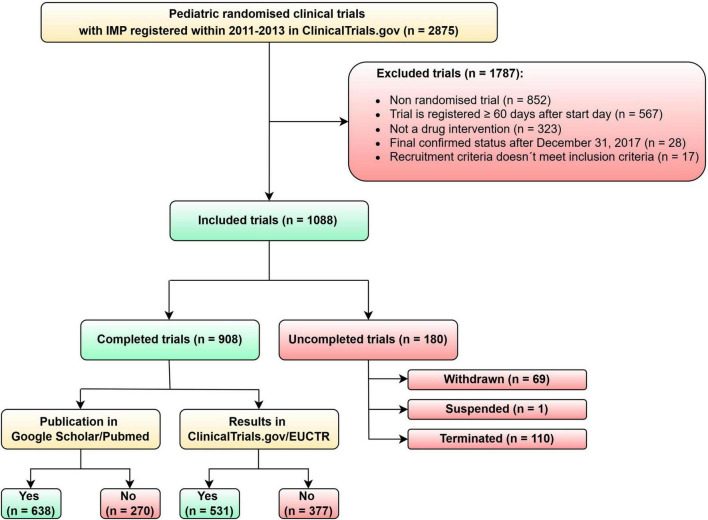
Flow diagram of pediatric clinical trials selected for analysis.

The dataset contains data from 2875 pediatric clinical trials. We identified 1787 pediatric clinical trials that did not meet the inclusion criteria for our analysis and 1088 that fulfilled inclusion criteria and were included in the analysis. As shown in Figure 1, there were five reasons for excluding trials. The most frequent reason was that the trial was not randomized (*n* = 852; 47.68%). Other reasons why trials were excluded: the trial was registered more than 60 days after the trial start day (*n* = 567; 37.73%), the trial was not a drug interventional trial (*n* = 323; 18.07%), final confirmed status was after December 31, 2013 (*n* = 28; 1.57%) and the last reason was that recruitment criteria did not meet inclusion criteria (*n* = 17; 0.95%). This last exclusion criterion was selected if the start date was earlier than 1.1.2011 or multiple exclusion criteria were present in one trial. The number of clinical trials that were included in our analysis is 1088.

### 3.2 Characteristics of the included pediatric RCTs

Of all included trials, 528 (48.53%) had an academic primary sponsor, and 491 (45.13%) trials had an industrial primary sponsor. The most represented age groups were combined (children + adult) (*n* = 558; 51.29%) and mixed age (*n* = 354; 32.54%) groups, followed by the preterm/newborn/infant group (*n* = 99; 9.10%) (Table 1). Certain infectious and parasitic diseases (17.10%) and diseases of the respiratory system were among the most common medical areas of interest (15.90%) (Table 2).

**TABLE 1:** Characteristics of completed and uncompleted pediatric RCTs.

	All included trials (*N* = 1,088)		Completed trials (*N* = 908)		Uncompleted trials (*N* = 180)		
	**n**	**%**	**n**	**%**	**n**	**%**	***p*-value**
**Year registered**							0.4839
2011	397	36.49	338	37.22	59	32.78	
2012	394	36.21	327	36.01	67	37.22	
2013	297	27.30	243	26.76	54	30.00	
All	1,088	100.00	908	100.00	180	100.00	
**Age of participant**							0.2294
Preterm, newborn, infant	99	9.10	77	8.48	22	12.22	
Toddler and preschool	26	2.39	24	2.64	2	1.11	
School age	26	2.39	23	2.53	3	1.67	
Adolescent	25	2.30	18	1.98	7	3.89	
Mixed age	354	32.54	296	32.60	58	32.22	
Combined^a^	558	51.29	470	51.76	88	48.89	
All	1,088	100.00	908	100.00	180	100.00	
**Funding**							0.0441
Academic	528	48.53	427	47.03	101	56.11	
Industry	491	45.13	425	46.81	66	36.67	
Other^b^	69	6.34	56	6.17	13	7.22	
All	1,088	100.00	908	100.00	180	100.00	
**Masking**							0.3138
Open Label	254	23.35	206	22.69	48	26.67	
Single	85	7.81	69	7.60	16	8.89	
Double	173	15.90	145	15.97	28	15.56	
Triple	178	16.36	144	15.86	34	18.89	
Quadruple	398	36.58	344	37.89	54	30.00	
All	1,088	100.00	908	100.00	180	100.00	
**Patients enrolled**							< 0.0001
<50	356	32.72	210	23.13	146	81.11	
51–100	216	19.85	202	22.25	14	7.78	
101–500	338	31.07	322	35.46	16	8.89	
501–1,000	97	8.92	94	10.35	3	1.67	
>1,000	81	7.44	80	8.81	1	0.56	
All	1,088	100.00	908	100.00	180	100.00	
**Planned sample size**							0.0022
<50	238	21.88	182	20.04	56	31.11	
51–100	236	21.69	201	22.14	35	19.44	
101–500	422	38.79	349	38.44	73	40.56	
501–1,000	102	9.38	93	10.24	9	5.00	
>1,000	86	7.90	79	8.70	7	3.89	
UNK	4	0.37	4	0.44			
All	1,088	100.00	908	100.00	180	100.00	
**Phase**							0.0077
I	58	5.33	47	5.18	11	6.11	
II	291	26.75	231	25.44	60	33.33	
III	417	38.33	370	40.75	47	26.11	
IV	183	16.82	148	16.30	35	19.44	
UNK	139	12.78	112	12.33	27	15.00	
All	1,088	100.00	908	100.00	180	100.00	

*^a^*Children and adults.

*^b^*Government-funded trials.

**TABLE 2:** Disease categories addressed in 1,088 included pediatric RCTs.

Condition category	n	%
Certain infectious and parasitic diseases	186	17.10
Diseases of the respiratory system	173	15.90
Endocrine, nutritional, and metabolic diseases	108	9.93
Mental and behavioral disorders	84	7.72
Symptoms, signs, and abnormal clinical and laboratory findings, not elsewhere classified	67	6.16
Diseases of the skin and subcutaneous tissue	51	4.69
Diseases of the nervous system	50	4.60
Diseases of the eye and adnexa	43	3.95
Diseases of the digestive system	41	3.77
Diseases of the blood and blood-forming organs and certain disorders involving the immune mechanism	35	3.22
Congenital malformations, deformations, and chromosomal abnormalities	33	3.03
Injury, poisoning and certain other consequences of external causes	33	3.03
Neoplasms	33	3.03
Certain conditions originating in the perinatal period	32	2.94
Diseases of the circulatory system	24	2.21
Diseases of the musculoskeletal system and connective tissue	22	2.02
Pregnancy, childbirth, and the puerperium	21	1.93
Diseases of the genitourinary system	18	1.65
External causes of morbidity and mortality	11	1.01
Diseases of the ear and mastoid process	10	0.92
Factors influencing health status and contact with health services	7	0.64
Metabolic disorders	2	0.18
Unknown	2	0.18
Diseases of oral cavity, salivary glands and jaws	1	0.09
Other and unspecified disorders of the circulatory system	1	0.09

#### 3.2.1 Uncompleted pediatric RCTs

One hundred eighty (16.54%) of included clinical trials (*n* = 1088) were uncompleted, with 61.11% (*n* = 110) terminated, 38.33% (*n* = 69) withdrawn, and one trial suspended.

The most common reasons for incompletion were patient accrual (32.22%), company decision (12.22%), and informative decision (11.67%) (Table 3), e.g., safety and efficacy reasons or interim analysis revealed a negative effect. Many clinical trials did not have a reason for their incompletion stated in the registry, and the reason was not traceable even in publications (22.78%).

**TABLE 3:** Reasons for clinical trial incompletion.

Uncompleted trials (*N* = 180)	n	%
Patient accrual	58	32.22
Nonreported or unclear	41	22.78
Company/business decision	22	12.22
Informative termination^a^	21	11.67
Funding issue	13	7.22
Conduct problems^b^	10	5.56
Regulatory issue^c^	8	4.44
Principle investigator left	7	3.89

*^a^*Includes changes in standard of care and safety or efficacy findings or interim analysis revealed a negative effect.

*^b^*Includes technical difficulties and logistical issues.

*^c^*Includes issues with institutional review board or other regulatory body.

Combined age, mixed age, and preterm/newborn/infant age groups were most often represented (Table 1) in uncompleted clinical trials.

The relationship between reasons for trial incompletion and the number of enrolled patients is well illustrated in Table 4. Out of a total of 180 uncompleted trials, 92 of them were not published anywhere. The number of patients enrolled in these studies was 3267.

**TABLE 4:** Reasons for trial incompletion and number of enrolled patients in terminated and suspended trials.

Terminated and suspended (*N* = 111)^a^	Number of enrolled patients				
**Reason for a trial incompletion**	**<50**	**51–100**	**101–500**	**501–1,000**	**>1,000**
	**n (%)**	**n (%)**	**n (%)**	**n (%)**	**n (%)**
Patient accrual	40 (36.04)	4 (3.60)	1 (0.90)	1 (0.90)	–
Informative termination	9 (8.11)	5 (4.50)	5 (4.50)	–	–
Nonreported or unclear	9 (8.11)	2 (1.80)	4 (3.60)	–	1 (0.90)
Company/business decision	6 (5.41)	1 (0.90)	4 (3.60)	1 (0.90)	–
Conduct problems	4 (3.60)	1 (0.90)	1 (0.90)	1 (0.90)	–
Principle investigator left	3 (2.70)	1 (0.90)	–	–	–
Regulatory issue	3 (2.70)	–	1 (0.90)	–	–
Funding issue	3 (2.70)	–	–	–	–

*^a^*Definition for a withdrawn trial is: a clinical trial stopped early, before enrolling its first participant. This is a reason for not including them in this table.

Statistically significant was also a difference in funding (*p* = 0.0441) (Table 1), with 101 (56.11%) uncompleted clinical trials receiving funding from an academic source, 66 (36.67%) uncompleted trials from industry, and 13 (7.22%) trials receiving funds from another source (e.g., government-funded studies).

The number of pediatric patients enrolled in uncompleted clinical trials was 9,904. This number was counted for terminated and suspended clinical trials only as no patients were recruited in the clinical trials with withdrawn status.

A statistically significant difference was found between completed and uncompleted trials in planned sample size (*p* = 0.0022), the number of patients enrolled (*p* < 0.0001), and the phase of the clinical trial (*p* = 0.0077).

### 3.3 Access to the results in completed pediatric RCTs

**FIGURE 2:**
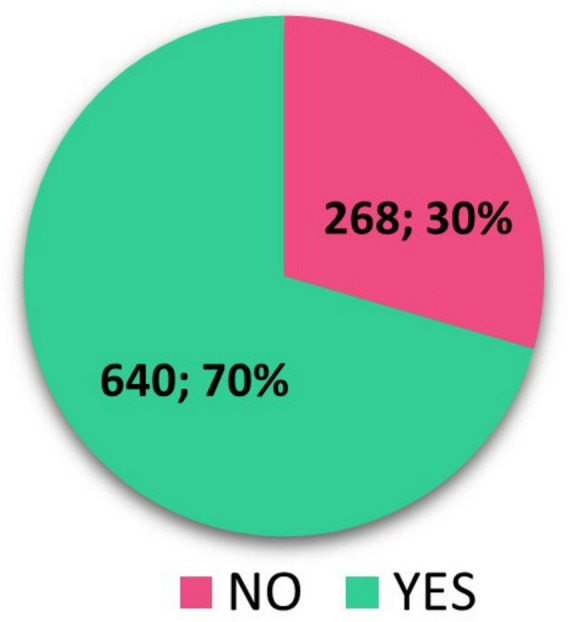
Scientific publication found in ClinicalTrials.gov/EUCTR and/or in Google Scholar/PubMed.

Overall, we have identified 908 completed pediatric RCTs in our research, where we searched for published results of these clinical trials. We focused on results recorded directly in clinical trial registries (ClinicalTrials.gov and EUCTR) and publications with clinical trial results in peer-reviewed journals. We have identified 531 (58.48%) completed pediatric RCTs that have posted results in ClinicalTrials.gov + EUCTR and 377 (41.52%) completed pediatric RCTs with no results in registers. The number of completed pediatric RCTs with trial results published in peer-reviewed journals found in Google Scholar/PubMed and ClinicalTrials.gov/EUCTR as well was 640 (70%) (Figure 2).

Table 5 presents an analysis of the relationship between the posting of clinical trial results on ClinicalTrials.gov and EUCTR (the European Clinical Trials Registry) and the presence of corresponding scientific publications identified in ClinicalTrials.gov, EUCTR, Google Scholar, or PubMed.

**TABLE 5:** Results posted in ClinicalTrials.gov + EUCTR and Scientific publications found in ClinicalTrials.gov + EUCTR and/or in Google Scholar/PubMed.

Results posted in ClinicalTrials.gov + EUC	Scientific publication found in ClinicalTrials.gov + EUCTR and/or in Google Scholar/PubMed		
	**No**	**Yes**	**Total**
No	125 13.77%	252 27.75%	377 41.52%
Yes	143 15.75%	388 42.73%	531 58.48%
Total	268 29.52%	640 70.48%	908 100.00%

The data are divided into categories based on whether results were posted in the registries and whether a corresponding scientific publication was found. These findings are dependent parameters, as the Chi-Square test indicates (*p*-value is 0.0427):

1. Results Not Posted, No Scientific Publication: 125 clinical trials (13.77%) neither had results posted in ClinicalTrials.gov or EUCTR nor had any scientific publication associated with them.
2. Results Not Posted, Scientific Publication Found: 252 clinical trials (27.75%) did not have results posted in the registries but were found to have a corresponding scientific publication.
3. Results Posted, No Scientific Publication: 143 clinical trials (15.75%) had their results posted in the registries but did not have a corresponding scientific publication.
4. Results Posted, Scientific Publication Found: 388 clinical trials (42.73%) had both their results posted in the registries and a corresponding scientific publication.

For 504 (55.51%) completed clinical trials, the registry included a reference to a scientific publication with the clinical trials’ results. The remaining 404 (44.49%) completed clinical trials were without any reference to scientific publication. Table 6 shows the relevance of the publications found in the registry to the clinical trial’s outcomes, with 86.9% being relevant and 13.1% being classified as non-relevant.

**TABLE 6:** References to publications listed in the ClinicalTrials.gov registry and publication’s relevance.

	n	%
No links to publications	404	44.49
Links to publications presented	504	55.51
Relevant	438	86.90
Not relevant	66	13.10

The age of the participants (*p* = 0.0255), funding (*p* < 0.0001), masking (*p* = 0.0234), the patient enrolled (*p* = 0.0096), and phase (*p* < 0.0001) were found to be significant determinants of (not)posting results in ClinicalTrials.gov and EUCTR (Supplementary Table S1). In multivariate analysis, evaluating all factors together, funding, phase, and patient enrollment remained the factors associated with posting results in ClinicalTrials.gov and EUCTR (Table 7). Pediatric RCTs with industrial funding have a 3.3 times greater chance of being posted results than academic RCTs. Phase I clinical trials have a 78% lower chance of having published trial results in ClinicalTrials.gov/EUCTR compared to Phase III clinical trials. Clinical trials with more than 1,000 enrolled patients have a 63% lower chance of being posted in ClinicalTrial.gov and EUCTR compared to clinical trials with < 50 patients.

**TABLE 7:** Significant factors associated with posting results in ClinicalTrials.gov + EUCTR.

Parameter	OR (95% CI)	*p*-value
Funding industry vs. academic	3.332 (2.344; 4.737)	<0.0001
Phase I vs. III	0.223 (0.109; 0.456)	<0.0001
Patient enrolled > 1,000 vs. <50	0.369 (0.204; 0.669)	0.0010

There is a statistically significant difference between completed clinical trials with and without scientific publication found in the age of the participants (*p* = 0.0096), the patient enrolled (*p* < 0.0001) and planned sample size (*p* < 0.0001) (Supplementary Table S2). Funding was evaluated as probably not statistically significant in this case. In Supplementary Table S2, we can see a higher percentage of scientific publications in academic pediatric RCT in comparison with industry sponsors.

In multivariate analysis, masking, participant age, and patient enrollment were significant factors in evaluating all factors together (Table 8). Pediatric RCTs with more than 1000 enrolled patients have a 4.8x higher chance of publishing scientific publications than clinical trials with < 50 patients. RCTs involving school-age participants have a 75% lower chance of having found their scientific publication compared to RCTs involving only preterm, newborn, or infant participants.

**TABLE 8:** Significant factors associated with having a scientific publication found in ClinicalTrials.gov + EUCTR and/or in Google Scholar/PubMed.

Parameter	OR (95% CI)	*p*-value
Masking double vs. open label	0.596 (0.368; 0.965)	0.0354
Patient enrolled 51–100 vs. < 50	1.929 (1.266; 2.939)	0.0022
Patient enrolled 101–500 vs. < 50	2.354 (1.580; 3.509)	<0.0001
Patient enrolled 501–1,000 vs. < 50	2.481 (1.388; 4.432)	0.0022
Patient enrolled > 1,000 vs. < 50	4.847 (2.351; 9.990)	<0.0001
Age of participant combined vs. preterm, newborn, infant	0.361 (0.185; 0.704)	0.0028
Age of participant school age vs. preterm, newborn, infant	0.245 (0.085; 0.706)	0.0092

#### 3.3.1 Time to publication of results in the completed pediatric RCTs

For completed clinical trials, the median time from the end of the trial to the first publication of results in ClinicalTrials.gov/EUCTR was 21 months (min/max: −35.93/119.77). There was no statistically significant difference between parameters in completed clinical trials that posted results within 12 months or after 12 months in ClinicalTrials.gov/EUCTR.

The median time from the end of the clinical trial to the publication of the results in peer-reviewed journals was almost 27 months (min/max: −17.97/108.79) for completed clinical trials). After focusing on statistically significant differences in some parameters of completed studies, in multivariate analysis, funding, phase, and patient enrollment remained the significant factors associated with publication within 12 months (Table 9). Trials with industrial funding have a 79% lower chance of being published within 12 months than academic clinical trials. In some cases, results or publications in peer-reviewed journals were released before the clinical trial was completed. Reasons for that might be that the sponsor or investigator had submitted the results from interim analysis into registries or to a journal.

**TABLE 9:** Significant factors associated with the trial’s result publishing within 12 months.

Parameter	OR (95% CI)	*p*-value
Funding industry vs. academic	0.210 (0.119; 0.372)	<0.0001
Patient enrolled > 1000 vs. < 50	2.675 (1.229; 5.824)	0.0132
Phase II vs. I	0.312 (0.120; 0.806)	0.0162
Phase IV vs. I	0.157 (0.055; 0.451)	0.0006

#### 3.3.2 Access to the results in uncompleted pediatric RCTs

Reporting of results of uncompleted pediatric RCTs was analyzed, and 62 (56.36%) of 110 terminated RCTs reported the results in registries. Almost 23% (*n* = 25) of uncompleted RCTs had a relevant publication with clinical trial results found in a peer-reviewed journal. Three publications did not mention that the clinical trial was terminated early, yet they reported the results without any warning. In contrast, the remaining articles consistently highlighted the limitations.

## 4 Discussion

Our analysis focused on RCTs in the pediatric population with drug intervention completed or finished by the end of year 2017.

Despite efforts to raise awareness of this issue through previously published work (4), the number of uncompleted RCTs in the pediatric population is increasing. Pica et al. (16) found that 10% of RCTs were not completed in the previous review period. According to our findings, the number of incomplete RCTs actually increased to 16.5% in the subsequent follow-up period. The most common reason for incompletion was insufficient patient recruitment, which remains the same identified factor in the previous period by Pica and Bourgeois (16). In more than half of the discontinued trials, a total of ten thousand patients were recruited. Despite the short duration of the clinical trials we analyzed, this figure is quite alarming. It is evident, that the conduct of pediatric clinical trials deserves increased attention. Although the current legal framework states the necessity of publishing results, just over half of the completed clinical trials had their results listed in registries (ClinicalTrials.gov and EUCTR), and only 20% had results published in registries within 12 months of clinical trial completion. A significant proportion of trials had results registered four years or more after the clinical trial completion date (17%). Interestingly, 70% of completed clinical trials had results traceable to peer-reviewed journals. Only 15% of pediatric RCTs had published results in scientific articles within 12 months. Similar to results published in registries, we found a high number of clinical trials (20%) with the first publication of results in scientific journals four years or more after clinical trial completion.

Although our analysis categorized pediatric trials consistently with previous research, it is important to recognize that the pediatric population encompasses a wide range of developmental stages, from pre-term neonates and infants to adolescents. These different age groups can vary substantially in terms of enrollment feasibility, consent and assent requirements, pharmacokinetics, pharmacodynamics, and disease characteristics. Such differences may influence both trial design and outcomes, including recruitment speed, treatment efficacy, and safety profiles. Due to limited age-specific detail in registry data, further subgroup analysis was not feasible, but we highlight this as a relevant consideration for future studies (3.1 Trial Characteristics). We decided to use the register ClinicalTrials.gov as the source data for our analysis. This register includes a vast number of clinical trials from around the world, not just from the United States, making it more comprehensive and globally relevant compared to the EUCTR register, which is focused only on clinical trials within the European Union. This broader scope provided a wider data set for our research. The data structure and presentation are detailed, the register’s environment is user-friendly, and the choice of filters allowed us to obtain the data set that we needed for our analyses.

Failure to complete a clinical trial always causes significant financial, time, and ethical losses. The reasons for early termination can be heterogeneous (3.2.1 Uncompleted pediatric RCTs). The most frequently cited reason in registries was low patient recruitment. This reason has remained unchanged for many years and has been mentioned in previous trials as a major challenge in pediatric clinical trials (4, 16, 20, 21). Compared to the adult population, this particular barrier may be explained by the low number of pediatric patients for a given disease and the process of obtaining informed consent, which has been more complex in children (22, 23). Although new legislation in the EU is already fully in place, allowing a child to be enrolled in a trial on the basis of consent from only one parent, in some countries, both parents still need to give informed consent, which can have a negative impact on overall trial recruitment. Public funding agencies, institutional review boards, and ethics committees should also require investigators to provide empirical evidence to support the feasibility of achieving the target sample size within an acceptable recruitment period: published or at least registered pilot clinical trials that include obtaining informed consent from participants are likely to provide the best evidence of the feasibility of the clinical trial protocol and provide the most realistic estimates for participant recruitment (24).

There is also increasing recognition of the importance of involving children and families in recruitment, consent, and study design (3, 25). This approach can strongly contribute to the feasibility and clinical trial completion.

To overcome the problem of small sample sizes in children, significant advances have been made in the development of statistical methods and in the collaboration of dedicated international groups and networks of pediatric clinical trials that pool their data and resources. Including the European Clinical Research Infrastructure Network (ECRIN) in the preparation and conduct of multicenter and multinational clinical trials, particularly those involving pediatric populations, represents a significant advantage. All these factors can improve the feasibility of the clinical trial.

Commercial RCTs are more likely to be completed. This is consistent with previous research performed by Pica and Bourgeois (16) And Rees et al. (26). One of the factors could be the interest of the pharmaceutical company to complete the clinical trial and get their product on the market. Financial considerations may play an important role here when in commercial clinical trials, the investigators are used to get fees for their scientific work, which increases the motivation of the trialists. Moreover, pharmaceutical companies are more familiar with involving patient advocates in the planning and organizing of clinical trials and usually have more developed technical infrastructure. On the other hand, with academic clinical trials, research is often funded by public funds and foundations, which have time and financial limits that can be challenging to meet.

A previous research study published in Pediatrics that reviewed interventional pediatric clinical trials registered with ClinicalTrials.gov between 2008 and 2010 indicates that 29.8% of completed trials remained unpublished in peer-reviewed journals (16). In our research, which loosely followed the above study with an analysis of completed trials registered in the same registry between 2011 and 2013, 30% of pediatric RCTs had no published results in scientific journals and 42% in registries (3.3 Access to the results in completed pediatric RCTs). Similar findings are reported in another study where 50% of the trials registered in EUCTR as completed or terminated by 2017 had published results in the registry (9). The same collective of authors from The Bennett Institute for Applied Data Science at the University of Oxford further addressed this topic and created websites (27) with tools that clearly show the number of published and unpublished clinical trial results by sponsors in the registries. In other words, these monthly updated trackers monitor the compliance of every individual sponsor from the ranks of pharmaceutical companies, universities, hospitals or foundations with FDAAA 2007 (FDAAA TrialsTracker) (28) and EU rules (EU Trials Tracker) (29). Brewster and colleagues found that only 23.5% of completed pediatric clinical trials registered between 2007 and 2020 reported results on the ClinicalTrials.gov registry, and 38.8% of the clinical trials were published in a peer-reviewed journal (4).

Our analysis also shows that industry-funded trials were more likely to have their results published in registries and less likely to be published in peer-reviewed journals than academic clinical trials. The disparity in the publication of results between non-commercial and commercial sponsors is highlighted in the Joint Letter by the European Commission, EMA, and HMA from April 2019. The letter first points to the overall compliance with the publication rules, with 68% of completed RCTs publishing their results. Further evaluation of this group by the sponsor shows a disproportion between industry sponsors and academic sponsors in reporting compliance (77% for commercial sponsors vs. 23% for academic sponsors) (30).

It is likely that academic sponsors are unaware of their obligations or do not have administrative procedures or sources in place to highlight non-compliance. To be compliant with the legislative requirements and good clinical practice, the sponsor is responsible for reporting the clinical trial results to the registry. However, in practice, in the case of hospitals acting as sponsors of an academic clinical trial, the principal investigator often becomes responsible and may not be sufficiently informed of the reporting obligation (9). Pfizer has reported that the preparation of results summaries requires 4–60 h, and it is possible that the academic sponsor doesn’t have administrative sources available to ensure reporting on time (31). Another significant reason could be the incentive structure. Commercial sponsors, such as pharmaceutical companies, are often motivated to publish results due to the financial implications of drug development and market approval. Regulatory bodies (FDA or EMA) enforce strict guidelines, and failure to comply may lead to penalties or delays in product approval. Furthermore, academic researchers may focus on publishing in high-impact journals to advance their careers or secure further funding. As a result, registry submissions, which may not carry the same academic recognition, are sometimes deprioritized. Caution should be exercised when interpreting the results of prematurely terminated and, therefore, underpowered pediatric RCTs. Nevertheless, their results can still be considered valid and could potentially contribute valuable pilot data for future studies. In addition, these findings could influence systematic reviews and meta-analyses (24).

There is no doubt that it is necessary to report the results of studies in registries according to the instructions and requirements of each registry. On the other hand, we believe that publishing results in peer-reviewed journals is more readable and accessible to the scientific community. But for the patient community, it is hardly accessible, mainly because of non-open access articles or the requirement to be a health professional. Ideally, the clinical trial sponsor would share the positive or negative results of the trial with the scientific and lay public community in both forms (results in the registry + scientific publication). However, almost 14% of completed pediatric RCTs did not publish their results in either a registry or a scientific journal. This completely contradicts the ethical commitment to participants who agreed to contribute to scientific progress in the expectation that their participation would produce publicly available knowledge (19). Specifically, academic and other academic sponsors should be encouraged to publish the results of their trials in registries in order to maximize their valuable contribution to meeting public health needs and to the advancement of clinical research, especially where there are fewer commercial interests (30).

As mentioned previously, timely reporting of clinical trial results is also a legal requirement. Our results show a median time for results publication of 21 months (3.3.1 Time to publication of the result in completed pediatric RCTs). Anderson’s study focusing on compliance with result reporting at ClinicalTrials.gov describes a median time of 17 months (31). While prompt dissemination of clinical trial data ensures that researchers, healthcare providers, and policy-makers can access the most current evidence, enabling informed decision-making and fostering evidence-based clinical practices. In addition, timely reporting may reduce the risk of redundant research (32).

We believe that the public ranking of sponsors’ performance in registry reporting, carried out through the work of the Bennett Institute’s team of experts, and the dissemination of information related to clinical trial registration and publication of clinical trial results provided by the AllTrials.net initiative (33) is meaningful. This may represent one way to encourage sponsors (e.g., to avoid reputational damage) not to underestimate the reporting of study results. Another approach to improving compliance with EU and US regulations on reporting study results would be for national regulatory authorities to conduct open public audits of compliance and regularly fine or withhold funding from those violating these ethical and legal rules.

In summary, our analysis maps the current trends of a number of pediatric clinical trials, highlighting significant challenges in their completion and publication of their results related to this vulnerable population. The unique ethical, regulatory, and logistical hurdles associated with pediatric research—such as recruitment difficulties, ethical considerations related to informed consent, and the complexity of pediatric clinical trial design—contribute to a higher rate of uncompleted RCTs compared to adult RCTs. Furthermore, our findings indicate substantial delays or outright omissions in the reporting of pediatric RCT results, both in clinical trial registries and in peer-reviewed journals. This underreporting compromises the transparency and availability of evidence necessary to make a reasonable decision in clinical practice and policy in pediatric care.

The discrepancies in reporting, particularly among academic sponsors, suggest that further incentives or enforcement mechanisms may be required to ensure the timely dissemination of clinical trial results. Addressing these barriers is crucial to improving pediatric research outcomes, fostering greater trust in the clinical trial process, and ensuring that children benefit from advancements in medical science. Future efforts should focus on enhancing regulatory oversight, streamlining clinical trial processes, and encouraging broader compliance with reporting rules to advance the quality and impact of pediatric clinical research.

## Data Availability

The original contributions presented in this study are included in this article/Supplementary material, further inquiries can be directed to the corresponding author.

## Supplementary material

Supplementary Table S1 Posting of results in ClinicalTrials.gov + EUCTR.

Supplementary Table S2 Scientific publication found in ClinicalTrials.gov/EUCTR and/or in Google Scholar/PubMed.

Supplementary Table S3 Protocol. Prospective protocol for data extraction, classification, and analysis.
