# Supplementary material for: Exploring the publication gap in pediatric randomized clinical trials: completed vs. uncompleted pediatric clinical trials
Source: Front Med (Lausanne). 2025 May 30;12:1590125. doi: 10.3389/fmed.2025.1590125 (PMC12163232; doi:10.3389/fmed.2025.1590125)
Supplement: Supplementary Table S1 — Posting of results in ClinicalTrials.gov + EUCTR. [file Table_1.docx]

|  |  |
| --- | --- |

**S1 Table.** **Posting of results in ClinicalTrials.gov + EUCTR**

|  | **Completed trials**  **(N=908)** | |  | **Not posted results**  **(N=377)** | | **Posted results (N=531)** | |  |
| --- | --- | --- | --- | --- | --- | --- | --- | --- |
|  | **n** | **%** |  | **n** | **%** | **n** | **%** | **p-value** |
| **Year registered** |  |  |  |  |  |  |  | 0.2135 |
| 2011 | 338 | 37.22 |  | 131 | 34.75 | 207 | 38.98 |  |
| 2012 | 327 | 36.01 |  | 148 | 39.26 | 179 | 33.71 |  |
| 2013 | 243 | 26.76 |  | 98 | 25.99 | 145 | 27.31 |  |
| All | 908 | 100.00 |  | 377 | 100.00 | 531 | 100.00 |  |
| **Age of participants** |  |  |  |  |  |  |  | 0.0255 |
| Preterm, newborn, infant | 77 | 8.48 |  | 38 | 10.08 | 39 | 7.34 |  |
| Toddler and preschool | 24 | 2.64 |  | 8 | 2.12 | 16 | 3.01 |  |
| School age | 23 | 2.53 |  | 5 | 1.33 | 18 | 3.39 |  |
| Adolescent | 18 | 1.98 |  | 3 | 0.80 | 15 | 2.82 |  |
| Mixed age | 296 | 32.60 |  | 116 | 30.77 | 180 | 33.90 |  |
| Combined^a^ | 470 | 51.76 |  | 207 | 54.91 | 263 | 49.53 |  |
| All | 908 | 100.00 |  | 377 | 100.00 | 531 | 100.00 |  |
| **Funding** |  |  |  |  |  |  |  | <0.0001 |
| Academic | 427 | 47.03 |  | 234 | 62.07 | 193 | 36.35 |  |
| Industry | 425 | 46.81 |  | 112 | 29.71 | 313 | 58.95 |  |
| Other^b^ | 56 | 6.17 |  | 31 | 8.22 | 25 | 4.71 |  |
| All | 908 | 100.00 |  | 377 | 100.00 | 531 | 100.00 |  |
| **Masking** |  |  |  |  |  |  |  | 0.0234 |
| Open Label | 206 | 22.69 |  | 106 | 28.12 | 100 | 18.83 |  |
| Single | 69 | 7.60 |  | 29 | 7.69 | 40 | 7.53 |  |
| Double | 145 | 15.97 |  | 57 | 15.12 | 88 | 16.57 |  |
| Triple | 144 | 15.86 |  | 55 | 14.59 | 89 | 16.76 |  |
| Quadruple | 344 | 37.89 |  | 130 | 34.48 | 214 | 40.30 |  |
| All | 908 | 100.00 |  | 377 | 100.00 | 531 | 100.00 |  |
| **Patients enrolled** |  |  |  |  |  |  |  | 0.0096 |
| <50 | 210 | 23.13 |  | 92 | 24.40 | 118 | 22.22 |  |
| 51-100 | 202 | 22.25 |  | 90 | 23.87 | 112 | 21.09 |  |
| 101-500 | 322 | 35.46 |  | 122 | 32.36 | 200 | 37.66 |  |
| 501-1000 | 94 | 10.35 |  | 29 | 7.69 | 65 | 12.24 |  |
| >1000 | 80 | 8.81 |  | 44 | 11.67 | 36 | 6.78 |  |
| All | 908 | 100.00 |  | 377 | 100.00 | 531 | 100.00 |  |
| **Planned sample size** |  |  |  |  |  |  |  | 0.3921 |
| <50 | 182 | 20.04 |  | 80 | 21.22 | 102 | 19.21 |  |
| 51-100 | 201 | 22.14 |  | 86 | 22.81 | 115 | 21.66 |  |
| 101-500 | 349 | 38.44 |  | 138 | 36.60 | 211 | 39.74 |  |
| 501-1000 | 93 | 10.24 |  | 32 | 8.49 | 61 | 11.49 |  |
| >1000 | 79 | 8.70 |  | 39 | 10.34 | 40 | 7.53 |  |
| UNK | 4 | 0.44 |  | 2 | 0.53 | 2 | 0.38 |  |
| All | 908 | 100.00 |  | 377 | 100.00 | 531 | 100.00 |  |
| **Phase** |  |  |  |  |  |  |  | <0.0001 |
| I | 47 | 5.18 |  | 32 | 8.49 | 15 | 2.82 |  |
| II | 231 | 25.44 |  | 89 | 23.61 | 142 | 26.74 |  |
| III | 370 | 40.75 |  | 118 | 31.30 | 252 | 47.46 |  |
| IV | 148 | 16.30 |  | 73 | 19.36 | 75 | 14.12 |  |
| UNK | 112 | 12.33 |  | 65 | 17.24 | 47 | 8.85 |  |
| All | 908 | 100.00 |  | 377 | 100.00 | 531 | 100.00 |  |

^a^children and adults

^b^government-funded trials

**S2 Table. Scientific publication found in ClinicalTrials.gov/EUCTR and/or in Google Scholar/PubMed.**

|  | **Completed trials**  **(N=908)** | |  | **No publication found**  **(N=268)** | | **Publication found (N=640)** | |  |
| --- | --- | --- | --- | --- | --- | --- | --- | --- |
|  | **n** | **%** |  | **n** | **%** | **n** | **%** | **p-value** |
| **Year registered** |  |  |  |  |  |  |  | 0.5054 |
| 2011 | 338 | 37.22 |  | 92 | 34.33 | 246 | 38.44 |  |
| 2012 | 327 | 36.01 |  | 101 | 37.69 | 226 | 35.31 |  |
| 2013 | 243 | 26.76 |  | 75 | 27.99 | 168 | 26.25 |  |
| All | 908 | 100.00 |  | 268 | 100.00 | 640 | 100.00 |  |
| **Age of participants** |  |  |  |  |  |  |  | 0.0096 |
| Preterm, newborn, infant | 77 | 8.48 |  | 12 | 4.48 | 65 | 10.16 |  |
| Toddler and preschool | 24 | 2.64 |  | 5 | 1.87 | 19 | 2.97 |  |
| School age | 23 | 2.53 |  | 10 | 3.73 | 13 | 2.03 |  |
| Adolescent | 18 | 1.98 |  | 6 | 2.24 | 12 | 1.88 |  |
| Mixed age | 296 | 32.60 |  | 78 | 29.10 | 218 | 34.06 |  |
| Combined | 470 | 51.76 |  | 157 | 58.58 | 313 | 48.91 |  |
| All | 908 | 100.00 |  | 268 | 100.00 | 640 | 100.00 |  |
| **Funding** |  |  |  |  |  |  |  | 0.1416 |
| Academic | 427 | 47.03 |  | 114 | 42.54 | 313 | 48.91 |  |
| Industry | 425 | 46.81 |  | 139 | 51.87 | 286 | 44.69 |  |
| Other | 56 | 6.17 |  | 15 | 5.60 | 41 | 6.41 |  |
| All | 908 | 100.00 |  | 268 | 100.00 | 640 | 100.00 |  |
| **Masking** |  |  |  |  |  |  |  | 0.0635 |
| Open Label | 206 | 22.69 |  | 56 | 20.90 | 150 | 23.44 |  |
| Single | 69 | 7.60 |  | 15 | 5.60 | 54 | 8.44 |  |
| Double | 145 | 15.97 |  | 54 | 20.15 | 91 | 14.22 |  |
| Triple | 144 | 15.86 |  | 49 | 18.28 | 95 | 14.84 |  |
| Quadruple | 344 | 37.89 |  | 94 | 35.07 | 250 | 39.06 |  |
| All | 908 | 100.00 |  | 268 | 100.00 | 640 | 100.00 |  |
| **Patients enrolled** |  |  |  |  |  |  |  | <0.0001 |
| <50 | 210 | 23.13 |  | 92 | 34.33 | 118 | 18.44 |  |
| 51-100 | 202 | 22.25 |  | 58 | 21.64 | 144 | 22.50 |  |
| 101-500 | 322 | 35.46 |  | 84 | 31.34 | 238 | 37.19 |  |
| 501-1000 | 94 | 10.35 |  | 23 | 8.58 | 71 | 11.09 |  |
| >1000 | 80 | 8.81 |  | 11 | 4.10 | 69 | 10.78 |  |
| All | 908 | 100.00 |  | 268 | 100.00 | 640 | 100.00 |  |
| **Planned sample size** |  |  |  |  |  |  |  | <0.0001 |
| <50 | 182 | 20.04 |  | 73 | 27.24 | 109 | 17.03 |  |
| 51-100 | 201 | 22.14 |  | 61 | 22.76 | 140 | 21.88 |  |
| 101-500 | 349 | 38.44 |  | 97 | 36.19 | 252 | 39.38 |  |
| 501-1000 | 93 | 10.24 |  | 25 | 9.33 | 68 | 10.63 |  |
| >1000 | 79 | 8.70 |  | 9 | 3.36 | 70 | 10.94 |  |
| UNK | 4 | 0.44 |  | 3 | 1.12 | 1 | 0.16 |  |
| All | 908 | 100.00 |  | 268 | 100.00 | 640 | 100.00 |  |
| **Phase** |  |  |  |  |  |  |  | 0.0935 |
| I | 47 | 5.18 |  | 19 | 7.09 | 28 | 4.38 |  |
| II | 231 | 25.44 |  | 80 | 29.85 | 151 | 23.59 |  |
| III | 370 | 40.75 |  | 97 | 36.19 | 273 | 42.66 |  |
| IV | 148 | 16.30 |  | 41 | 15.30 | 107 | 16.72 |  |
| UNK | 112 | 12.33 |  | 31 | 11.57 | 81 | 12.66 |  |
| All | 908 | 100.00 |  | 268 | 100.00 | 640 | 100.00 |  |

^a^children and adults

^b^government-funded trials
