# Supplementary material for: Exploring the publication gap in pediatric randomized clinical trials: completed vs. uncompleted pediatric clinical trials
Source: Front Med (Lausanne). 2025 May 30;12:1590125. doi: 10.3389/fmed.2025.1590125 (PMC12163232; doi:10.3389/fmed.2025.1590125)
Supplement: Supplementary Table S2 — Scientific publication found in ClinicalTrials.gov/EUCTR and/or in Google Scholar/PubMed. [file Table_2.docx]

Protocol: Exploring the Publication Gap in Pediatric Randomized Clinical Trials: Completed vs. Uncompleted Clinical Trials

Finalized: 25.11.2021

Primary outcomes:

- Prevalence of completed pediatric RCTs which have posted results in the register (Clinicaltrials.gov and EUCTR) and their characteristics
- Prevalence of pediatric RCTs which have results published in peer-reviewed journals

Secondary outcomes:

- Prevalence of uncompleted pediatric RCTs
- Reasons for discontinuation
- Time to publication of results (in registries, in peer-reviewed journals)
- Number of enrolled pediatric patients in uncompleted patients

Trial selection and data extraction from ClinicalTrials.gov:

*Inclusion criteria*

- Randomized controlled trials (RCTs) – intervervention: drug
- Trials which include children – 0-17 let
- Trials registered between 1.1.2011 – 31.12.2013 which were completed or discontinued by 31.12.2017 (Recruitment status – completed, terminated, withdrawn, suspended).

*Exclusion criteria:*

- not a pediatric trial
- not a drug intervention trial
- non randomized trial
- recruitment criteria don´t meet inclusion criteria
- final confirmed status after December 31, 2017

Advance search in Clinicaltrials.gov using the following searching fields:

- study type – interventional study

- intervention/treatment - drug

- status recruitment – suspended, terminated, completed, withdrawn

- age group – child (birth -17)

- study registered (first posted) - 1.1.2011 – 31.12.2013

- primary completion date – from 0 – to 31.12.2017

Definitions:

- Uncompleted trial: recruitment status =’suspended’, ‘withdrawn’, and ‘terminated’
- Completed trial: recruitment status =’completed’
- Funding source:
- Industry-funded = primary sponsor is the industry
- Academia = primary sponsor university, hospital, foundation
- Other = government-funded trials
- Age category:
- Pretern, newborn, infant = 0-1 year
- Toddler and preschool = 2-5 years
- School age = 6-11 years
- Adolescent = 12-17 years
- Mixed ages = mixed age children
- Combined = children + adults
- Published: Publication as a peer-reviewed journal article

Publication search:

Review ClinicalTrials.gov to find links to publications – relevant publications:

- NCT is mentioned in the article (abstract or full text)

- and the article is published after the study is completed

If a publication was not listed in the publication field of the registry entry (our case n=404) or was not relevant, the publication was searched on the basis of the NCT or other identification number (e.g. EudraCT). Google Scholar and PubMed sites were used for this purpose using NCT number (or EudraCT number), trial title, author names, institutions, and study keywords. Articles are linked to the corresponding trials based on a comparison of the trial data provided in the registry entry and in the abstract or full manuscript, when necessary.

Results search:

Review ClinicalTrials.gov to find results of completed RCTs. Studies with no published results in Clinicaltrials.gov were searched in register EUCTR. If results were found in EUCTR we added them to the studies with results found in Clinicaltrials.gov.

Analyses:

Characteristics of the different trial groups:

- NTC Number
- Age of participants
- Condition cathegory
- Masking
- Planned sample size and actual enrollment
- Trial phase
- Year registered, year completed
- Primary funding source

Prevalence of incompletion, reason for incompletion, Factors associated with uncompleted trials (sample size, sponsor type,…), number of enrolled pediatric patients enrolled into uncompleted trials.

Prevalence of non-publication of pediatric RCT results: *all completed trials*

- Number of pediatric RCTs which have publication, number of pRCTs which posted results, number of pRCTs that have neither been published nor published results
- Factors associated with nonpublication (type of sponsor, sample size, masking,..)

Time to publication in a peer-reviewed journal:

- Time from the end of the trial to the first publication found in Google Scholar/PubMed (in months)
- Time of the first publication of completed trials after the end of the trial (after 6, 12, 24, 36, 48 or more than 48 months)

Time to publication results in registries ClinicalTrials.gov/EUCTR:

- Time from the end of the trial to the first publication found in ClinicalTrials.gov/EUCTR (in months)
- Time of the first publication of completed trials after the end of the trial (after 6, 12, 24, 36, 48 or more than 48 months)
